# Peptides-Based Vaccine MP3RT Induced Protective Immunity Against *Mycobacterium Tuberculosis* Infection in a Humanized Mouse Model

**DOI:** 10.3389/fimmu.2021.666290

**Published:** 2021-04-26

**Authors:** Wenping Gong, Yan Liang, Jie Mi, Zaixing Jia, Yong Xue, Jie Wang, Lan Wang, Yusen Zhou, Shihui Sun, Xueqiong Wu

**Affiliations:** ^1^ Tuberculosis Prevention and Control Key Laboratory/Beijing Key Laboratory of New Techniques of Tuberculosis Diagnosis and Treatment, Institute for Tuberculosis Research, 8^th^ Medical Center, Chinese People's Liberation Army General Hospital, Beijing, China; ^2^ Graduate School, Hebei North University, Zhangjiakou, China; ^3^ State Key Laboratory of Pathogen and Biosecurity, Beijing Institute of Microbiology and Epidemiology, Beijing, China

**Keywords:** *Mycobacterium tuberculosis*, epitope peptide, vaccine, Th1-type immune responses, protective efficacy, humanized mice

## Abstract

**Background:**

Tuberculosis (TB) is still a global infectious disease that seriously threatens human beings. The only licensed TB vaccine Bacille Calmette-Guérin (BCG)’s protective efficacy varies significantly among populations and regions. It is very urgent to develop more effective vaccines.

**Methods:**

In this study, eleven candidate proteins of *Mycobacterium tuberculosis* were selected to predict peptides with high-affinity binding capacity for the HLA-DRB1*01:01 molecule. The immunodominant peptides were identified with the enzyme-linked immunospot assay (ELISPOT) and linked in silico to result in a novel polypeptide vaccine in *Escherichia coli* cells. The vaccine’s protective efficacy was evaluated in humanized and wild-type C57BL/6 mice. The potential immune protective mechanisms were explored with Enzyme-linked Immunosorbent Assay (ELISA), flow cytometry, and ELISPOT.

**Results:**

Six immunodominant peptides screened from 50 predicted peptides were used to construct a new polypeptide vaccine named MP3RT. After challenge with *M. tuberculosis*, the colony-forming units (CFUs), lung lesion area, and the number of inflammatory cells in humanized mice rather than wild-type mice vaccinated with MP3RT were significantly lower than these in mice immunized with PBS. The humanized mice vaccinated with MP3RT revealed significant increases in IFN-γ cytokine production, IFN-γ^+^ T lymphocytes, CD3^+^IFN-γ^+^ T lymphocytes, and the MP3RT-specific IgG antibody.

**Conclusions:**

Taken together, MP3RT is a promising peptides-based TB vaccine characterized by inducing high levels of IFN-γ and CD3^+^IFN-γ^+^ T lymphocytes in humanized mice. These new findings will lay a foundation for the development of peptides-based vaccines against TB.

## Introduction

As an ancient disease, tuberculosis (TB) has been a threat to human beings for thousands of years (1). Even today, with advanced technology, the number of deaths caused by TB still ranks first among the top ten infectious diseases (2). According to the Global Tuberculosis Report 2020 released by the World Health Organization (WHO), 10 million people developed TB and 1.2 million died in 2019 (3). Even more worrying is that Corona Virus Disease 2019 (COVID-19) has significantly impacted the TB epidemic and response. Current studies have reported the potential impact of the COVID-19 pandemic on global TB deaths and suggested that the TB mortality could increase to the levels seen in 2015 or even 2012 (3–5).

Vaccination is the best way to stop TB infection. Bacille Calmette-Guérin (BCG), the only licensed vaccine against TB infection, has been approved for neonatal vaccination in TB high-burden countries, which has made an outstanding contribution to controlling the incidence of TB in children. A previous modeling study estimated that BCG vaccination at birth could reduce TB deaths by 16.5%, but delays might increase TB deaths by 0.2% (6). These data indicate that avoiding BCG shortages and increasing BCG coverages at birth is an effective way to reduce global pediatric TB mortality. However, the duration of BCG protection is only 10-15 years, which is why the BCG vaccine has limited efficacy against pulmonary TB in adults (7).

It is urgent to develop a more effective vaccine to make up for the shortcomings of BCG. Pipelines for new TB vaccines are progressing with more than 25 vaccine candidates evaluated in clinical trials (2). Almost all of these TB candidates belong to first (live attenuated vaccines or inactivated vaccines), second (subunit vaccines), or third (DNA vaccines) generation vaccines. The latest generation of TB vaccine formulations is the development of peptide-based vaccines that emerged in recent years. Peptide-based vaccines consist of the immunodominant peptides of proteins recognized by T or B lymphocytes triggering T and B cell-mediated immune responses (8).

*Mycobacterium tuberculosis*, the pathogen of TB, is an intracellular parasitic bacterium, and the host’s removal or killing of *M. tuberculosis* mainly depends on macrophages and T lymphocytes. It is the widely accepted view that CD4^+^ T cells play an essential role in mycobacterial clearance. The recognition of CD4^+^ T cells and antigen-presenting cells (APCs) is limited by the major histocompatibility complex II molecule (MHC II). As a new discipline developed in recent years, immunoinformatics provides a possibility for predicting immunogenic T-cell peptides of *M. tuberculosis* that can be used to develop a peptide-based vaccine (9). According to the Immune Epitope Database (IEDB), up to 63% of epitopes are related to the MHC class II molecule. Previous data have demonstrated that the MHC class II molecule plays an essential role in bridging peptides’ presentation and activating T-helper 1 (Th1) immune response (10–13).

Furthermore, the selection of animal models is crucial for the evaluation of peptides-based vaccines, because the recognition of this vaccine and host T lymphocytes depends on MHC restriction. In our previous study, we have constructed a humanized C57BL/6 mice (HLA-A11^+/+^ DRB1*01:01^+/+^H-2-β2m^-/-^/IAβ^-/-^) for Chinese population (14). Herein, we selected this mouse model to develop a novel vaccine based on Th1-type peptides might help fight against TB infection. In this study, the immunodominant peptides were identified from the candidate Th1 peptides predicted by the IEDB database (http://www.iedb.org/) using an enzyme-linked immunospot (ELISPOT) array. The nucleotide sequences of these immunodominant peptides were linked in silico to product a novel polypeptide vaccine named MP3RT in *Escherichia coli*. The protective efficacy of the MP3RT vaccine was evaluated in humanized and wild-type C57BL/6 mice, and its potential mechanism was explored in splenocytes *in vitro*.

## Materials and Methods

### Ethics Statement

All of the experiments related to animals were performed following the Experimental Animal Regulation Ordinances principles established by the China National Science and Technology Commission. Mice were well cared during their living, and all protocols were approved by the Animal Ethical Committee of the 8th Medical Center of Chinese PLA General Hospital (Approved Number: 309201808171015). All animals were raised in a SPF laboratory in the 8th Medical Center of Chinese PLA General Hospital, and the *M. tuberculosis* virulent strain challenge experiments were conducted in a qualified negative pressure biosafety laboratory level-2 PLUS (negative pressure BSL-2 PLUS) in the 8th Medical Center of Chinese PLA General Hospital.

The collection of human peripheral blood mononuclear cells (PBMCs) in TB patients, volunteers with latent tuberculosis infection (LTBI), and normal volunteers were performed following the principles of the Ethical Review of Biomedical Research Involving Humans established by NHFPC and the Declaration of Helsinki established by the World Medical Association (WMA). All participants signed written informed consent. The clinical investigation related to PBMCs isolation was approved by the Medical Ethics Committee of the 8th Medical Center of Chinese PLA General Hospital (Approved Number: 2018ST011).

### Bacterial Strains and Plasmids

*M. tuberculosis* (H37Rv strain) were cultured on Lowenstein-Jensen culture medium (Baso Biotechnology Co., LTD., Zhuhai, Guangdong province, China) at 37°C for 28 days and isolated from grinding fluid following our previous study (15). *M. tuberculosis*’s number and viability were determined with colony-forming units (CFUs) assay (16). Besides, the plasmid pET32a (+) and *Escherichia coli* BL21 cells were purchased from the Wuhan Institute of Biotechnology (Wuhan, Hubei, China) to express the target gene *in vitro* according to our previous studies (13, 17, 18).

### Mice and Subjects

Female wild-type C57BL/6 mice at the age of 7-8 weeks were obtained from Vital River Laboratories (Beijing, China), and female humanized C57BL/6 mice (HLA-A11^+/+^DR1^+/+^H-2-β2m^-/-^/IAβ^-/-^) with similar weight and age were presented by professor Yusen Zhou of Beijing Institute of Microbiology and Epidemiology (Beijing, China) (14). Furthermore, this study included 37 patients with TB, 11 volunteers with LTBI, and 62 normal volunteers. The recruitment was carried out at the 8th Medical Center of Chinese PLA General Hospital between December 2019 and June 2020. The diagnoses of TB and LTBI and the inclusion and exclusion criteria for normal volunteers were followed with the Diagnosis for Pulmonary Tuberculosis (WS288-2017) established by the National Health and Family Planning Commission of China (NHFPC).

### HLA-DRB1*01:01 Binding Epitopes Prediction and Peptide Synthesis

Eleven mycobacterial antigens, including Mpt51, Mpt63, Mpt64, Mtb8.4, PPE18, PPE44, PPE68, RpfA, RpfB, RpfE, and TB10.4 (**Table 1**), were selected to predict the dominant epitopes restricted by HLA-DRB1*01:01 molecule. Their amino acid sequences were downloaded from the National Center for Biotechnology Information (NCBI, https://www.ncbi.nlm.nih.gov/) database. The obtained amino acid sequences were imported into the IEDB to predict the potential dominant epitopes with high binding affinity to the human HLA-DRB1*01:01 allele as previously described (20, 21). Seven MHC II binding methods such as IEDB recommended, Consensus method, Combinatorial library, NN-align (netMHCII-2.2), SMM-align (netMHCII-1.1), Sturniolo, and NetMHCIIpan were used to predict the potential dominant epitopes. The selection IEDB Recommended uses the Consensus approach, combining NN-align, SMM-align, Combinatorial library and Sturniolo if any corresponding predictor is available for the molecule. Otherwise, NetMHCIIpan is used. The Consensus approach considers a combination of any three of the four methods, if available, where Sturniolo as a final choice.

**Table 1:** The basic information about vaccine candidate proteins of *M. tuberculosis*.

Protein Name	Accession No.^a^		Locus_tag	Gene Name^b^		Length (aa)		Annotation^b^		Group^c^		Summary Information ^b^	
Mpt51	CCP46632		Rv3803c	* fbpD/fbpC1/mpb51/mpt51*		299		Secreted MPT51/MPB51 antigen protein FbpD		NA		One of the major proteins in the culture filtrate of Mycobacterium bovis BCG	
Mpt63	CCP44693		Rv1926c	*mpt63/mpb63*		159		Immunogenic protein Mpt63		III		Predicted possible vaccine candidate	
Mpt64	CCP44749		Rv1980c	*mpt64/mpb64*		228		Immunogenic protein Mpt64		II		Predicted possible vaccine candidate	
Mtb8.4	CCP43930		Rv1174c	*TB8.4*		110		Low molecular weight T-cell antigen TB8.4		II		Predicted to be an outer membrane protein and possible vaccine candidate	
PPE18	CCP43952		Rv1196	*PPE18*		391		PPE family protein PPE18		NA		Member of the *Mycobacterium tuberculosis* PPE family	
PPE44	CCP45569		Rv2770c	*PPE44*		382		PPE family protein PPE44		NA		Member of the *Mycobacterium tuberculosis* PPE family	
PPE68	CCP46702		Rv3873	*PPE68*		368		PPE family protein PPE68		I		A peptide-based vaccine candidate	
RpfA	CCP43615		Rv0867c	*rpfA*		407		Possible resuscitation-promoting factor RpfA		I		Predicted possible vaccine candidate	
RpfB	CCP43759		Rv1009	*rpfB*		362		Probable resuscitation-promoting factor RpfB		I		Predicted possible vaccine candidate	
RpfE	CCP45243		Rv2450c	*rpfE*		172		Probable resuscitation-promoting factor RpfE		I		Predicted possible vaccine candidate	
TB10.4	CCP43018		Rv0288	*esxH/cfp7/TB10.4*		96		Low molecular weight protein antigen 7 EsxH		I		Predicted possible vaccine candidate	

a The National Center for Biotechnology Information (NCBI, http://www.ncbi.nlm.nih.gov/). Data were retrieved on 3 Mar 2017.

b The Gene name, annotation, and summary information are based on the data deposited at the NCBI. Data were retrieved on 3 Mar 2017.

c The group is based on a previous study (See 19). The antigens are sorted by the qualitative score (Qual Total) and subsequently by the quantitative score (Quant Total). Group I includes all antigens with a qualitative score 8 and above, provided that the quantitative score is not lower than 12. The rest of the antigens having a qualitative score of 8 and those having a qualitative score of 7 and a quantitative score not lower than 9 were clustered into Group II. Group III included antigens with qualitative scores of 7 (and a quantitative score of 8) and 6 (with a quantitative score of 9 and up).

NA, not available.

The predicted peptides were synthesized *in vitro* by SBS Genetech Co., Ltd. (Beijing, China) using a solid-phase synthesis method. Briefly, FMOC (9-fluorenylmethyloxycarbonyl) is used to protect the α-amino group of amino acids, the peptide is synthesized with TETRAS™ Peptide Synthesizer and cleaved from the resin with TFA (trifluoroacetic acid). Finally, the peptide was purified by a high-performance liquid chromatography (HPLC) and analyzed by a mass spec-trometer (MS) to make its purity higher than 75%.

### Immunodominant Peptides Screening

The ELISPOT assay was performed to screen the immunodominant peptides according to our previous study (21). The differences were described as follows. Four groups of female humanized mice (five mice per group) were immunized with 5×10^6^ CFUs of inactivated *M. tuberculosis* in 100 μl complete Freund’s adjuvant (CFA, Cat. No.F5881, Sigma-Aldrich, Missouri, USA), 500 μg lysate of *M. tuberculosis* in 100 μl phosphate buffer solution (PBS), 500 μg lysate of *M. tuberculosis* in 100 μl CFA, and 500 μg lysate of *M. tuberculosis* in 100 μl incomplete Freund’s adjuvant (IFA, Cat. No.F5506, Sigma-Aldrich, Missouri, USA), respectively. Fourteen days post-immunization, mice in each group were sacrificed, the splenocyte suspension was centrifuged at 4 °C and 500 g for 5 min, and the supernatant was discarded. The pellet was resuspended gently with 10 ml of 1 × Red Blood Cell Lysis Buffer (Cat. No.00-4333, eBioscience, Shanghai, China) and incubated at room temperature for 5 min. After washing twice with PBS, the splenocytes’ concentration was adjusted to 3 × 10^5^/ml with Roswell Park Memorial Institute (RPMI) 1640 Medium (Cat. No. 8115240, Gibco, Shanghai, China). Then, the interferon-γ (IFN-γ)^+^ T cells were detected by a Mouse IFN-γ ELISOPT^PLUS^ (Cat. No. 3321-4APT-2, Mabtech AB, Nacka Strand, Sweden). In detail, 100 μl of splenocytes and 10 μl of candidate peptide (2 μg) were added into a well of 96-well ELISPOT plate and incubated at 37°C. Twenty-four hours later, the splenocytes in the ELISPOT plate were gently removed, and the plate was washed five times with PBS. Subsequently, 100μl of R4-6A2 labeled monoclonal antibody (1μg/ml) was added to the ELISPOT plate and incubated at room temperature for 2h. After washing five times with PBS, 100 μl of streptavidin-ALP diluted 1:1000 with PBS containing 0.5% fetal bovine serum (FBS) was added to the ELISPOT plate and incubated for one hour at room temperature. After washing five times with PBS, 100 μl substrate solution (BCIP/NBT-plus) filtered with a 0.45 μm filter was added to the ELISPOT plate and stopped color development by washing extensively in tap water. The number of spots forming cells (SFCs) in each well was determined with a CTL-S5 Versa ELISPOT Reader (CTL, Cleveland, OH, USA). The immunodominant peptide was defined with a stimulation index (SI) value great than two following our previous study (21).

### Preparation and Three-Dimensional (3D) Structure Prediction of the Recombinant Polypeptide

The amino acid sequences of the immunodominant peptides screened by ELISPOT assay were linked with GGGGS or AAY linker in silico. Their hydrophilicity, amphipathic regions, and antigenic index were analyzed using a bioinformatics software Lasergene Protein (DNASTAR, Inc., Madison, Wisconsin, USA). After codon optimization, the nucleotide sequence of the recombinant polypeptide was synthesized by the Wuhan Institute of Biotechnology (Wuhan, Hubei, China) and inserted into the pET32a (+) plasmid (NCOI and XhoI sites) to transform *E. coli* cells *in vitro*. The expression and purification of MP3RT were performed by the C-terminal 6-his tag following our previous studies (12, 17, 18). The endotoxin of the purified antigen MP3RT were removed with Toxin EraserTM (GenScript, Piscataway, NJ) following our previous study (13). Furthermore, the candidate vaccine’s 3D structure was predicted by using the SWISS-MODEL database (https://swissmodel.expasy.org/interactive) according to a previous description (22).

### Mice Immunization and Infection

**Figure 1:**
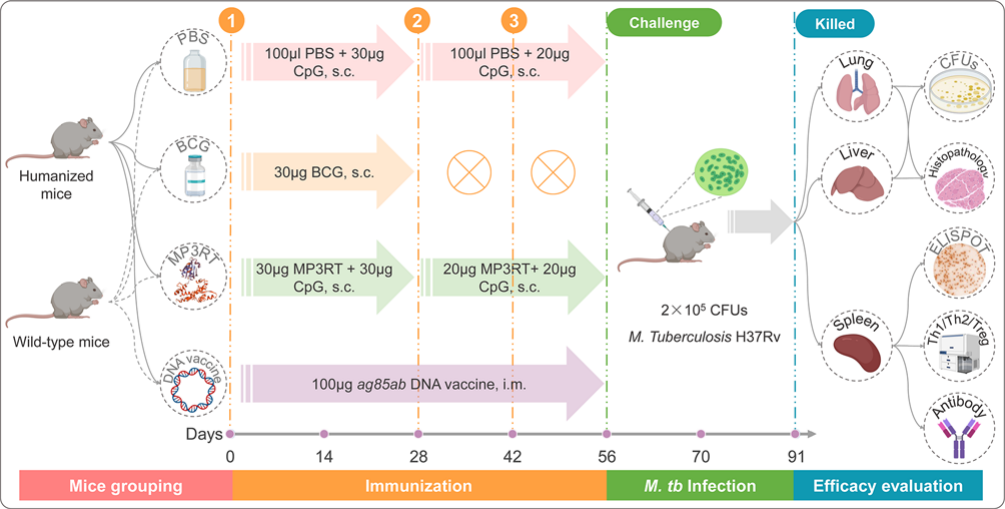
The flow chart of vaccination, infection, and evaluation.

The flow diagram of the immunization was presented in **Figure 1**. Three groups of humanized or wild-type mice (ten mice per group) were immunized subcutaneously with 30 μg CpG-ODN2395 adjuvants (Sangon, Shanghai, China) in 100 μl PBS, 30 μg BCG (Chengdu Institute of Biological Products Co., Ltd., Chengdu, Sichuan province, China) in 100 μl PBS, and 30 μg MP3RT combined with 30 μg CpG-ODN2395 adjuvants in 100 μl PBS, respectively. Twenty-eight and 42 days post primary immunization, the mice in PBS and MP3RT groups were boosted subcutaneously with 20 μg CpG-ODN2395 adjuvants in 100 μl PBS and 20 μg MP3RT in 100 μl PBS, respectively. The mice in the BCG group were not performed any booster immunization. Furthermore, to compare the protective efficacy of MP3RT and an *ag85ab* chimeric DNA vaccine whose immunogenicity and therapeutic effects have been confirmed in our previous study (23), ten humanized or wild-type mice were intramuscularly injected with 100 μg *ag85ab* DNA vaccine and boosted with the same dose on the 28^th^ and 42^nd^ days after the first immunization. Then, all of the mice were challenged with *M. tuberculosis* H37Rv strain (2 × 10^5^ CFUs) *via* tail vein injection on days 56 and killed on days 91.

### Mycobacterial Colony Counting

On the 91^st^ day after primary immunization, humanized or wild-type mice in each group were sacrificed, and their livers, lungs, and spleens were collected for efficacy evaluation. Briefly, the left lobe of the lung and half part of the liver were homogenized in normal saline (3 ml per organ), and the grinding fluid was diluted at 1:10, 1:100, and 1:1000 with normal saline. Then, 0.1 ml of the diluted solution was drawn from each diluted sample and inoculated on modified antibiotics-free Lowenstein-Jensen medium plates (Baso Biotechnology Co., LTD., Zhuhai, Guangdong province, China) in duplicate. Finally, CFUs were counted after incubating at 37°C for 28 days.

### Histopathology

The right lobe of the lung collected from each mouse was cut into blocks of 2.0 cm × 2.0 cm × 0.3 cm with ophthalmic scissors and then fixed overnight in formaldehyde solution (the required volume was 10 times of the sample volume). The tissue sample was dehydrated by ethanol with a concentration of 80%, 90%, 95%, and 100% (2 hours/time), embedded with paraffin, and cut into tissue sections with thickness 4-6 μm. Finally, these tissue sections were stained with hematoxylin and eosin (H&E) method following our previous studies (12, 13, 17, 18). Five tissue sections for each lung were independently observed by two researchers under a microscope of 40 × or 100 × (Olympus Corporation, Tokyo, Japan). The lesion area rate and the number of inflammatory cells were counted by Image-Pro Plus software (Version 6.0, Media Cybernetics, Inc: Bethesda, MD, USA).

### ELISPOT

ELISPOT experiment of mouse splenocytes: On 28 days after infection, humanized or wild-type mice were killed. Their spleens were collected to prepare splenocytes suspension according to the method described above. A volume of 100 μl splenocytes (3×10^6^/ml) was added into the wall of 96-well ELISPOT plate and incubated with 50 μl of PBS, 50 μl of MP3RT vaccine (60 μg/ml), or 50 μl of phytohemagglutinin (PHA, 60 μg/ml) at 37°C for 24h, respectively. The SFCs were determined according to the methods mentioned above.

ELISPOT experiment of PBMCs: The PBMCs were isolated from blood samples of TB patients, LTBI volunteers, and healthy volunteers using a Human peripheral blood mononuclear cell Isolation Kit (Solarbio, Beijing, China). The isolated PBMCs were added into the 96-well ELISPOT plate wall and incubated with 50 μl of PBS and 50 μl of MP3RT vaccine (60 μg/ml), respectively. Twenty-four hours later, SFCs were determined with a Human IFN-γ ELISpot^PRO^ kit (Cat. No. 3420-2APW-10, Mabtech AB, Nacka Strand, Sweden) following the manufactures’ introduction.

### Th1/Th2/Th17 Cytokines Analysis

The mice were killed on the 91^st^ day after the first immunization, and the spleens were collected for the preparation of splenocytes suspension following the above methods. A volume of 100μl splenocytes (3×10^6^/ml) and 50μl MP3RT vaccine (60 μg/ml) were incubated in a well of 96-well cell culture plate (Mabtech AB, Nacka Strand, Sweden) at 37°C for 48h. Then, the culture solution was transferred into a new tube and centrifugated at 500g for 10 min. Finally, the supernatant was gently transferred into another new tube, and the levels of interleukin -2 (IL-2), IL-4, IL-6, IL-10, IFN-γ, tumor necrosis factor -α (TNF-α), and IL-17A were detected by a Mouse Th1/Th2/Th17 Cytokine Kit (Cat. No. 560485, BD Biosciences, San Jose, CA, USA) following the manufacturer’s instruction.

### Flow Cytometry

Mouse splenocytes suspension (3×10^7^ cells/ml) was prepared according to the above-described method. The frequency of CD3^+^CD4^+^ T cells, CD3^+^IFN-γ^+^ Th1 cells, CD3^+^IL-4^+^ Th2 cells, and CD4^+^CD25^+^FoxP3^+^ regulatory T cells (Treg cells) was quantified with BD IntraSure™ kit (Cat. No. 641776, BD Biosciences, San Jose, CA, USA) according to the manufacturer’s instructions. This section contained two independent experiments, one to detect the frequency of CD3^+^IFN-γ^+^ Th1 cells and CD3^+^IL-4^+^ Th2 cells, and the other to detect the frequency of CD4^+^CD25^+^FoxP3^+^ Treg cells.

Experiment 1: Briefly, 100 µl suspension containing 3×10^6^ splenocytes was incubated with 10µl of MP3RT vaccine (1mg/ml) at 37°C for 4h. FITC Hamster Anti-Mouse CD3e (Cat. No.553061, BD Biosciences, San Jose, CA, USA) and Percp-cy 5.5 Rat Anti-Mouse CD8a (Cat. No. 561092, BD Biosciences, San Jose, CA, USA) were added into tubes for surface staining for 30 min at 4°C. The cell pellet was resuspended with 1ml of 1 × Fix/Perm Buffer solution (Cat. No. 51-9008100, BD Biosciences, San Jose, CA, USA), incubated at 4°C for 40 min protected from light, and washed twice with 1ml 1 × Perm/Wash Buffer solution at 4°C and 500g for 6 min. After that, the cells were incubated with 0.9 μl of PE Rat Anti-Mouse IFN-γ (Cat. No. 554412, BD Biosciences, San Jose, CA, USA), 2.4 μl of APC Rat Anti-Mouse IL-4 (Cat. No. 554436, BD Biosciences, San Jose, CA, USA), 1 μl of PE Rat IgG1 κ Isotype Control (Cat. No. 554685, BD Biosciences, San Jose, CA, USA), and 2.5μl of APC Rat IgG1 κ Isotype Control (Cat. No. 554686, BD Biosciences, San Jose, CA, USA) at 4°C for 40 min protected from light, respectively. After washing twice with 1 × Perm/Wash Buffer solution (Cat. No. 51-9008102, BD Biosciences, San Jose, CA, USA), the cells in each tube were resuspended with 350 μl PBS and analyzed on a flow cytometer (Beckman Coulter, Inc., Brea, CA, USA).

Experiment 2: Approximately 3×10^6^ prepared splenocytes in 100 µl of RPMI 1640 Medium were added into a tube and incubated with 1 µg of Ms CD16/CD32 Pure 2.4G2 (Cat. No. 553141, BD Biosciences, San Jose, CA, USA) at 4°C for 5min. Then, 2 μl of FITC-CD3 (Cat. No. 553061, BD Biosciences, San Jose, CA, USA), 2 μl of APC-CD4 (Cat. No. 553051, BD Biosciences, San Jose, CA, USA), and 5 μl of PE-CD25 antibodies (Cat. No. 553866, BD Biosciences, San Jose, CA, USA) were added into each sample tube. Then, 2μl of FITC-Ham IgG1 Kap (Cat. No. 553971, BD Biosciences, San Jose, CA, USA), 2μl of APC-Rat IgG2a Kap (Cat. No. 553932, BD Biosciences, San Jose, CA, USA), and 5μl of PE-Rat IgG1 Lam antibodies (Cat. No. 557078, BD Biosciences, San Jose, CA, USA) were added into isotype control tube, respectively. After incubation at 4°C for 40 min protected from light, the samples were washed with 2 ml PBS at 4°C and 500g for 6 min. The samples were incubated with 1ml of 1 × Fix/Perm Buffer solution at 4°C for 40 min protected from light, followed with twice washing with 1ml of 1 × Perm/Wash Buffer solution. Then, the cells in the sample tube and isotype control tube were incubated with 5 μl of BV4212-FoxP3 (Cat. No. 562996, BD Biosciences, San Jose, CA, USA) and 5 μl of Rat IgG2b Kpa ItCl BV4212 antibodies (Cat. No. 562603, BD Biosciences, San Jose, CA, USA) at 4°C for 40 min protected from light, respectively. After twice washing with 2 ml of 1 × Perm/Wash Buffer solution, the samples were resuspended with 350 μl PBS and analyzed on the flow cytometer (Beckman Coulter, Inc., Brea, CA, USA).

### Antibody Detection by Enzyme-Linked Immune Sorbent Assay (ELISA)

Blood samples were collected from the mice when they were sacrificed on day 91 after the first immunization. The collected blood sample was centrifuged at 1500rpm for 20 min, and then the supernatant was gently transferred into a new tube. The serum separated from each blood sample was linearly diluted (2 times) with PBS from a minimum dilution of 100 to a maximum dilution of 204800 to determine the optimum dilution by a Mouse ELISA Kit (Solarbio, Beijing, China) following the instruction given by the manufacturer. After that, the rest of the serum was diluted in the optimal dilution, and the levels of MP3RT specific IgG were determined by Goat anti-Mouse IgG/HRP (Cat. No. SE131, Solarbio, Beijing, China). Finally, the OD_450_ values of MP3RT specific IgG were detected with a microplate reader (Thermo Fisher Scientific, Shanghai, China).

### Statistical Analysis

All of the results in this study were performed using the GraphPad Prism 8 software (San Diego, CA, USA). The results of efficacy evaluation, pathological lesions, ELISA, ELISPOT on mice, cytokines, and flow cytometry were analyzed with Ordinary one-way ANOVA test or Kruskal-Wallis nonparametric test according to the data normality and homogeneity of variances. The ELISPOT experiment results on the samples collected from TB patients, LTBI, and healthy control were analyzed with an Unpaired *t*-test or nonparametric test (Mann Whitney test) according to the normality. The data was showed as mean ± standard error of the mean (SEM), and *P*-value < 0.05 was considered as a significant difference. Furthermore, in order to reduce the error caused by multiple comparisons, we also choose the method recommended by the GraphPad Prism 8 software for correction.

## Results

### Eight Immunodominant Peptides Identified With ELISPOT

**Figure 2:**
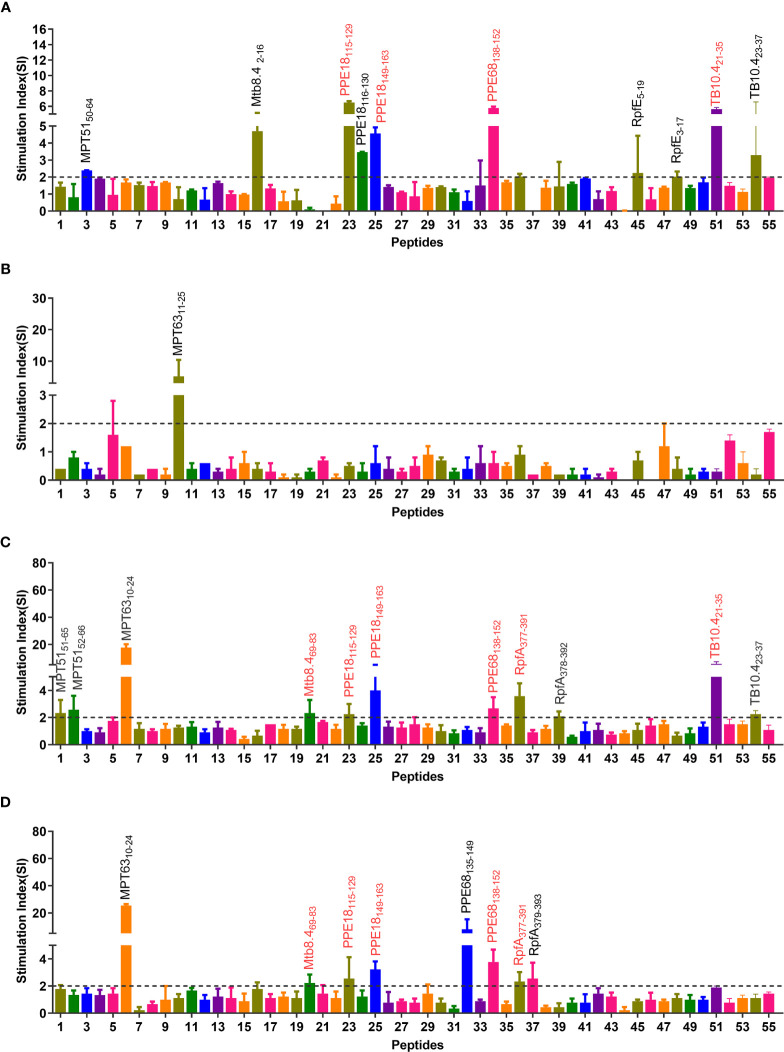
Immunodominant peptides screening *via* four independent ELISPOT experiments. Splenocytes obtained from humanized mice immunized with inactivated *M. tuberculosis* in CFA adjuvant **(A)**, lysate of *M. tuberculosis* in PBS **(B)**, lysate of *M. tuberculosis* in CFA adjuvant **(C)**, and lysate of *M. tuberculosis* in IFA adjuvant **(D)** were stimulated with MP3RT. The SFCs were analyzed by a CTL-S5 Versa ELISPOT Reader. The SI value of 55 peptides was shown as the ratio of SFCs in peptide stimulated cells and medium-stimulated cells. The detailed information of 55 peptides can be found in **Table S1**. SI >2 was considered positive and labeled in the figure. The immunodominant peptides included in the MP3RT vaccine were marked using a red text tag.

The epitopes predicted by the IEDB database were scored by the percentile rank, and the epitopes with scores below 10 in the rank were selected as the dominant Th1 epitopes (the lower the score, the higher the affinity). As a result, a total of 55 potential dominant epitopes were predicted (**Table S1**) and synthesized. After that, the potential immunodominant Th1 peptides were screened by ELISPOT assay, and the results showed that the SI values of eight peptides (MPT63_10-24_, Mtb8.4_69-83_, PPE18_115-129_, PPE18_149-163_, PPE68_138-152_, RpfA_377-391_, TB10.4_21-35_, and TB10.4_23-37_) were greater than 2 in at least two independent experiments (**Figure 2**). Our further study found that immunodominant peptide MPT63_10-24_ was a part of the signal peptide of MPT63 protein, indicating that these amino acid residues would be challenging to express *Escherichia coli* BL21 cells. We also observed that two immunodominant peptides TB10.4_21-35_ and TB10.4_23-37_ shared similar amino acid, and the SI value induced by peptide TB10.4_21-35_ was significantly higher than that of TB10.4_23-37_. Based on the reasons described above, both immunodominant peptides MPT63_10-24_ and TB10.4_23-37_ were not included in our further study.

### Sequence Optimization, Construction, and Expression of MP3RT Vaccine

**Figure 3:**
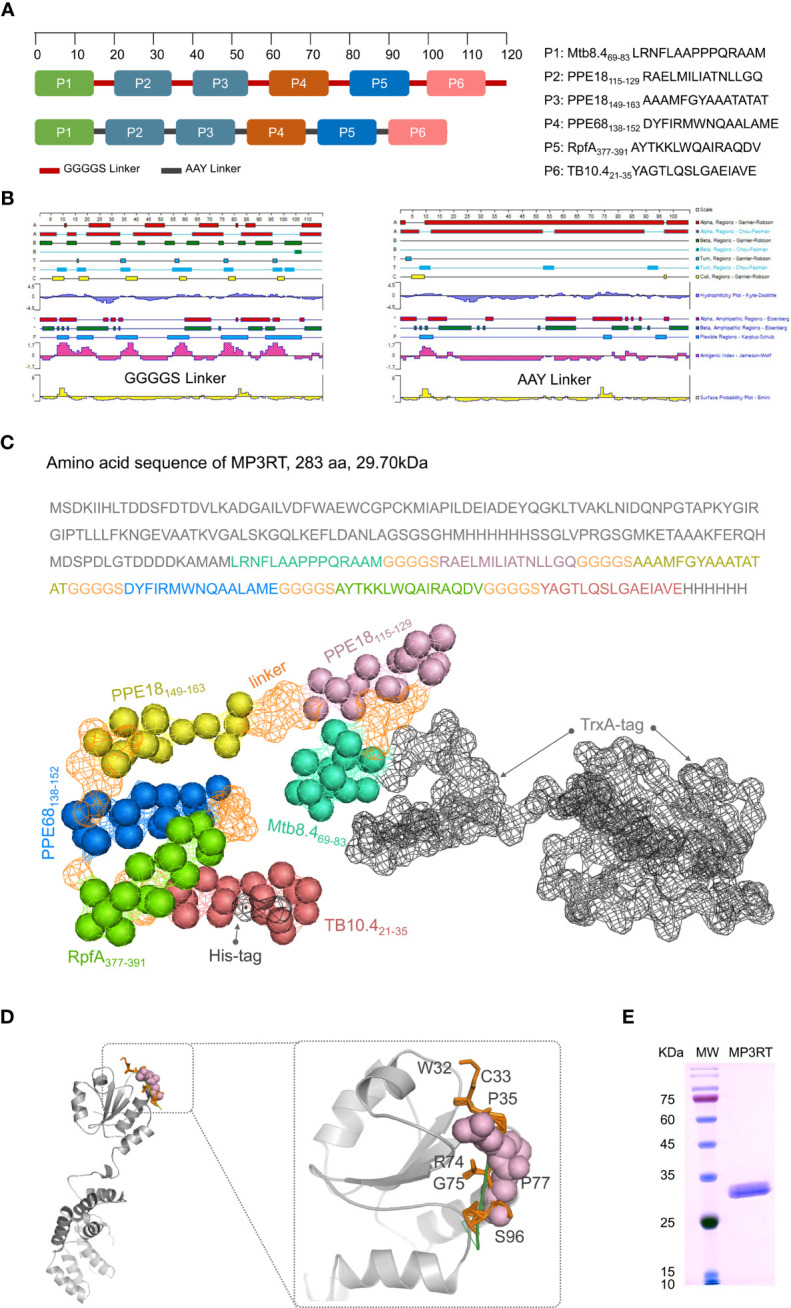
Construction and structural prediction of MP3RT vaccine. Six immunodominant peptides were linked with GGGGS or AAY linkers in silico **(A)**. Their antigenic index, surface probability, hydrophobicity, alpha regions, beta regions, and turn regions were assessed using Lasergene Protein software **(B)**. The length and molecular weight of MP3RT were 283aa and 29.70 kDa, respectively **(C)**. The 3D structure of MP3RT was presented in Cartoon, Spacefill, and Surface **(C, D)**. The MP3RT vaccine was prepared in *E. coli* cells and verified with polyacrylamide gel electrophoresis **(E)**.

It has been reported that optimizing peptide linkers with length, flexibility, and amino acid composition could improve the thermostability and activity of the displayed enzyme (24). The amino acid sequences of six immunodominant peptides were linked with flexible GGGGS linker or rigid AAY linker in silico to evaluate the hydrophilicity, antigenic index, and amphipathic regions (**Figure 3A**). The results indicated that the type of linker but not the order and continuity of these immunodominant peptides significantly affected the vaccine’s characteristics. Compared with rigid AAY linker, the flexible GGGGS linker significantly decreased the number of α-helices. It increased the antigen index, flexibility, and the number of secondary structures such as the β-sheet, β-turn, and random coils (**Figure 3B**). A recent study also reported that the flexible GGGGS linker provided the best structure and stability for fusion protein (25). Therefore, the GGGGS linker was selected to link the immunodominant peptides together with TrxA-tag and His-tag (**Figure 3C**). The three-dimensional (3D) structure prediction revealed that the MP3RT vaccine consisted of α-helixes and β-sheets, forming a hollow spindle structure (**Figures 3C, D**). Finally, a novel recombinant protein named as MP3RT (283aa in length and 29.7 kDa in molecular weight) was successfully expressed in *E. coli* cells following our previous studies (13, 21) (**Figure 3E**).

### Peptide-Based Vaccine MP3RT Elicited Protective Efficacy in Humanized Mice Rather Than Wild-Type Mice

**Figure 4:**
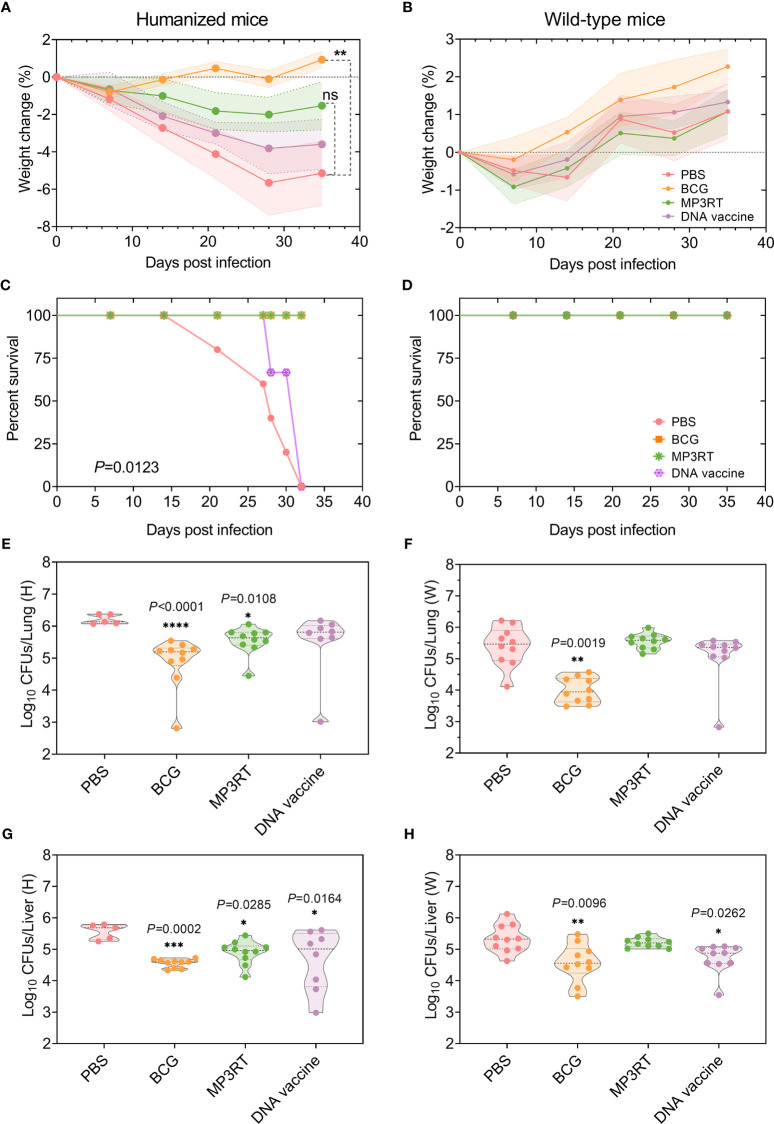
Protective efficacy of MP3RT vaccine in the mouse model. Humanized or wild-type C57BL/6 mice were immunized with PBS, BCG, MP3RT, and *ag85ab* DNA vaccine, respectively. On the 56 days after primary immunization, humanized or wild-type mice were challenged with *M. tuberculosis* H37Rv strain. The weight changes **(A, B)** and deaths **(C, D)** were recorded weekly or daily. Thirty-five days post-infection, mice were sacrificed. The left lobe of the lung **(E** or **F)** and half part of the liver **(G** or **H)** collected from humanized or wild-type mice were used for CFUs counting. The data were expressed as Log_10_ of CFUs and compared with one-way analysis of variance (ANOVA) or Kruskal-Wallis test according to the data normality and homogeneity of variances. All data were shown as mean + SEM (*n* = 5 in humanized mice vaccinated with PBS and *n* = 10 in other groups). *P*<0.05 was considered significantly different. *P < 0.05; **P < 0.01; *** P <0.001; ****P < 0.0001, ns, no significance.

To determine the protective efficacy of the MP3RT vaccine, the humanized mice and wild-type mice were immunized with PBS, BCG, MP3RT, and *ag85ab* DNA vaccine, respectively. Our results showed that mice’s weight in each group decreased during the early *M. tuberculosis* infection stage and increased in the late stage. Interestingly, we found that humanized mice’s weight has been on a downward trend and gradually began to rise until the 28th day after infection (**Figure 4A**). In contrast, the weight loss of humanized mice immunized with BCG (**Figure 4A**) and wild-type mice in each group appeared in the first week after infection and then began to increase gradually (**Figure 4B**). In addition, although we found that the weight of MP3RT-immunized mice recovered faster than the PBS negative control and DNA-immunized mice, there was no statistical difference between them (**Figure 4A**, *P* > 0.05). Only the weight of BCG-immunized mice recovered significantly better than that of PBS control group (**Figure 4A**, *P* < 0.01). These results suggested that the protection efficiency induced by MP3RT vaccination does not exceed that of BCG vaccine.

Moreover, five or two humanized mice vaccinated with PBS or DNA vaccine died after *M. tuberculosis* challenge (*P* = 0.0123, **Figure 4C**), respectively. On the contrary, none of the rest humanized mice and all wild-type mice died (**Figures 4C, D**). When the bacterial loads in the organs of humanized mice were compared, the CFUs in the lungs of mice immunized with BCG (*P* < 0.0001) or MP3RT (*P* = 0.0108) was less than that in the lungs of mice immunized with PBS (**Figure 4E**). The CFUs in the livers of mice immunized with BCG (*P* = 0.0002), MP3RT (*P* = 0.0285), or DNA vaccine (*P* = 0.0164) was remarkably lower than that of mice immunized with PBS (**Figure 4G**). As expected, the CFUs in the liver and lungs of MP3RT-immunized wild-type mice were not statistically different from the PBS group (**Figures 4F, H**). However, the CFUs in the lungs and livers collected from wild-type mice immunized with BCG (*P* = 0.0019, **Figure 4F**; *P* = 0.0096, **Figure 4H**) or DNA vaccine (*P* = 0.0262, **Figure 4H**) were lower than that of mice immunized with PBS. These results demonstrated that the MP3RT vaccine had an ability to reduce mycobacterial loads in humanized mice instead of wild-type mice. Moreover, the DNA vaccine may play a potential role in the prevention of TB.

### MP3RT Vaccination Significantly Reduced the Pathological Lesions

**Figure 5:**
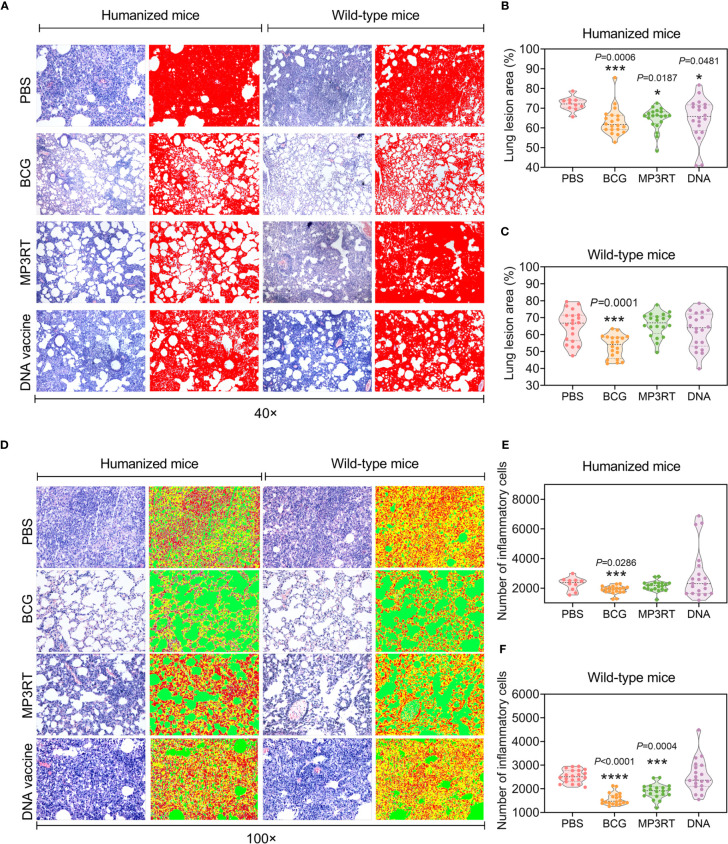
Histopathological characteristics of the lung from mice vaccinated with the MP3RT vaccine. The right lobe of the lung and the rest of the liver obtained from humanized or wild-type mice were used to perform H&E staining and analyzed with software. Each tissue section’s pathological changes were observed using a microscope with original magnification times of 40 × **(A)** and 100 × **(D)**. The lung lesion area of tissue section obtained from humanized **(B)** or wild-type mice **(C)** was marked as red and determined using Image-Pro Plus software. The inflammatory cells of the lung tissue section obtained from humanized **(E)** or wild-type mice **(F)** were determined using Image-Pro Plus software. The inflammatory cells were marked in red, alveoli were marked in green, and alveolar walls were marked in yellow. Observations and calculations were done independently by two researchers, and their calculation results were merged. Five tissue sections of each mouse were randomly selected for continuous observation and then took the average value. The results were statistically analyzed with one-way analysis of variance (ANOVA) or Kruskal-Wallis test according to the data normality and homogeneity of variances. All data were shown as mean + SEM (*n* = 10 in humanized mice vaccinated with PBS and *n* = 20 in other groups). *P *< 0.05 was considered significantly different. *P < 0.05; ***P < 0.001; ****P < 0.0001.

The reduction of mycobacterial loads was also reflected in the pathological assays. Humanized or wild-type mice’s lung lesions were observed under 40 × microscope and determined with Image-Pro Plus software (**Figure 5A**). The results suggested that the lung lesion areas of humanized mice vaccinated with BCG (*P* = 0.0006), MP3RT (*P* = 0.0187), or DNA vaccine (*P* = 0.0481) were significantly less than that of mice immunized with PBS (**Figure 5B**). The lung lesion area of wild-type mice vaccinated with BCG was less than that of wild-type mice immunized with PBS (*P* = 0.0001, **Figure 5C**). Furthermore, the number of inflammatory cells of the lungs obtained from humanized or wild-type mice were observed under 100 × microscope and counted with Image-Pro Plus software (**Figure 5D**). It was found that the number of inflammatory cells in the lungs collected from humanized mice vaccinated with BCG was less than that of mice in the PBS group (*P* = 0.0286, **Figure 5E**). The number of inflammatory cells in the lungs collected from wild-type mice vaccinated with BCG (*P* < 0.0001) and MP3RT (*P* = 0.0004) was less than that of wild-type mice in the PBS or DNA vaccine group (**Figure 5F**).

### MP3RT Induced an Increase in Number of IFN-γ^+^ T Lymphocytes in Mouse Splenocytes and Human PBMCs

**Figure 6:**
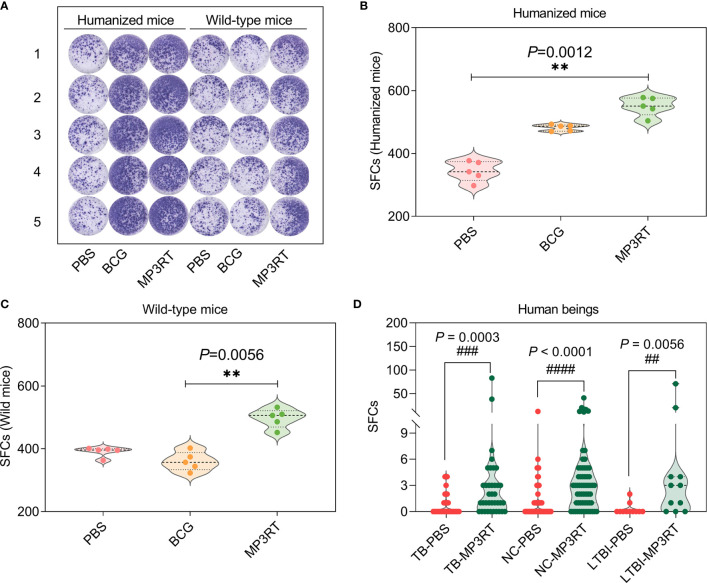
IFN-γ^+^ T lymphocytes detection with ELISPOT. The MP3RT vaccine was used to stimulate the PBMCs collected from humanized mice or wild-type mice vaccinated with PBS, BCG, and MP3RT *in vitro* **(A)**. The number of IFN-γ^+^ T lymphocytes (showed as SFCs) in humanized mice **(B)** and wild-type mice **(C)** were determined with a mouse ELISPOT kit. The data were analyzed with a one-way analysis of variance (ANOVA) or Kruskal-Wallis test according to the data normality and homogeneity of variances. All data were shown as mean + SEM (*n* = 5). *P* < 0.05 was considered significantly different. **, *P* < 0.01. Furthermore, the number of IFN-γ^+^ T lymphocytes in PBMCs collected from TB patients, LTBI volunteers, and normal controls (NC) were stimulated with the MP3RT vaccine *in vitro*. **(D)** The number of IFN-γ^+^ T lymphocytes were determined with a human ELISPOT kit. The data were analyzed with the Unpaired *t*-test or nonparametric test (Mann Whitney test) according to the normality. All data were shown as mean + SEM (*n* = 37, 11, and 62 in TB patients, LTBI volunteers, and normal controls, respectively). *P*<0.05 was considered significantly different. ^##^ *P* < 0.01; ^###^ *P* <0.001; ^####^ *P* < 0.0001.

To determine the IFN-γ secreting cells induced by MP3RT vaccine, ELISPOT assays were conducted in splenocytes of mice (**Figure 6A**) and PBMCs of human beings (the spot diagrams are not shown), respectively. The results showed that the number of IFN-γ^+^ T lymphocytes (showed as SFCs in **Figure 6**) of humanized mice (*P* = 0.0012, **Figure 6B**) or wild-type mice (*P* = 0.0056, **Figure 6C**) vaccinated with MP3RT was significantly higher than that of mice vaccinated with PBS. Similar results were observed in PBMCs collected from 37 TB patients, 11 LTBI volunteers, and 62 normal controls (**Figure 6D**). With the comparison of PBS stimulation, the stimulation of MP3RT resulted in a significantly higher levels of IFN-γ^+^ T lymphocytes in TB patients (*P* = 0.0003), persons with LTBI (*P* = 0.0056), and normal controls (*P* < 0.0001), respectively.

### A High Level of IFN-γ Induced by MP3RT Vaccination in Humanized Mice

**Figure 7:**
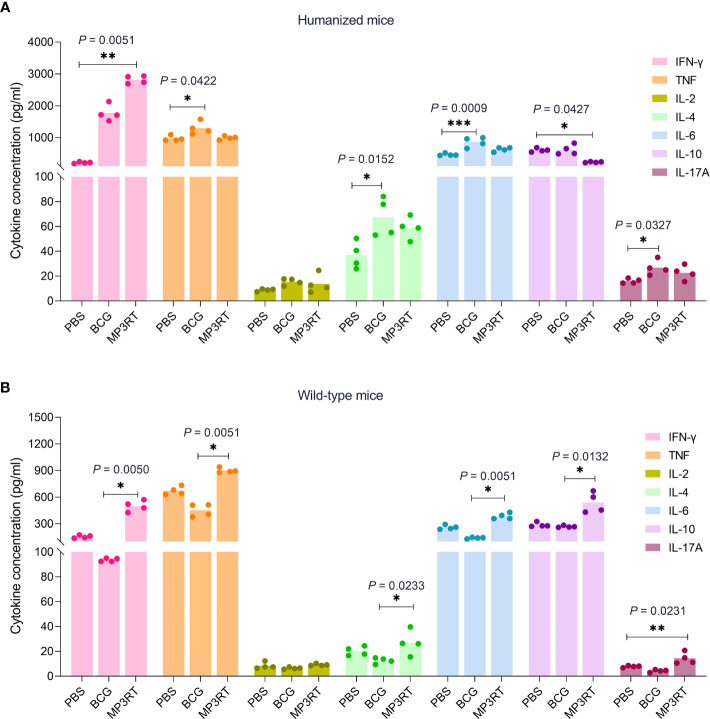
Cytokines. The splenocytes isolated from humanized mice **(A)** or wild-type mice **(B)** immunized with PBS, BCG, or MP3RT were stimulated with the MP3RT vaccine for 48 hours. The levels of IFN-γ, TNF-α, IL-2, IL-4, IL-6, IL-10, and IL-17A cytokines in the supernatant were detected with a Mouse Th1/Th2/Th17 Cytokine Kit. The differences were compared with the one-way analysis of variance (ANOVA) or Kruskal-Wallis test according to the data normality and homogeneity of variances. All data were shown as mean + SEM (*n* = 4). *P* < 0.05 was considered significantly different. *P < 0.05; **P < 0.01; ***P < 0.001.

To determine the cytokines profile induced by the MP3RT vaccine, the Th1/Th2/Th17 cytokines were detected with a Mouse Th1/Th2/Th17 Cytokine Kit. In humanized mice, the level of IFN-γ produced by splenocytes of MP3RT vaccinated mice was significantly higher than that of PBS vaccinated mice (*P* = 0.0051, **Figure 7A**). However, the level of IL-10 secreted by splenocytes of MP3RT vaccinated mice was significantly lower than that of PBS vaccinated mice (*P* = 0.0427, **Figure 7A**). Furthermore, the levels of TNF-α (*P* = 0.0422), IL-4 (*P* = 0.0152), and IL-6 (*P* = 0.0009), and IL-17A (*P* = 0.0327) secreted by splenocytes of BCG vaccinated mice were higher than those of PBS vaccinated mice (**Figure 7A**). In wild-type mice, the levels of IFN-γ (*P* = 0.0050), TNF-α (*P* = 0.0051), IL-4 (*P* = 0.0233), IL-6 (*P* = 0.0051), and IL-10 (*P* = 0.0132) secreted by mice vaccinated with MP3RT were significantly higher than those of mice vaccinated with BCG (**Figure 7B**). Moreover, the level of IL-17A secreted by wild-type mice vaccinated with MP3RT was significantly higher than that of mice vaccinated with PBS (*P* = 0.0231, **Figure 7B**). In contrast, there was no significant difference in the level of IL-2 among groups of humanized (**Figure 7A**) or wild-type mice (**Figure 7B**).

### Vaccination With MP3RT Induced a High Rate of Lymphocytes

**Figure 8:**
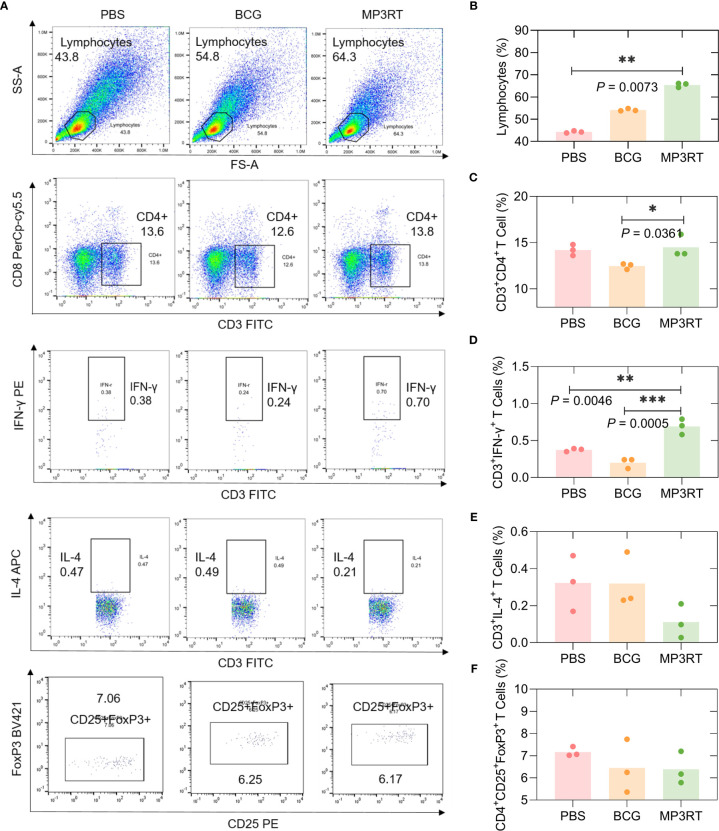
The frequency of lymphocytes, CD3^+^CD4^+^ T cells, CD3^+^IFN-γ^+^ T cells, CD3^+^IL-4^+^ T cells, and CD4^+^CD25^+^FoxP3^+^ Treg T cells in humanized mice. The splenocytes suspension was prepared, and the frequency of lymphocytes **(A, B)**, CD3^+^CD4^+^ T cells **(A, C)**, CD3^+^IFN-γ^+^ T cells **(A, D)**, CD3^+^IL-4^+^ T cells **(A, E)**, and CD4^+^CD25^+^FoxP3^+^ regulatory T cells **(A, F)** was quantified with a BD IntraSure™ kit. The differences in the frequency of cells among PBS, BCG, and MP3RT groups were analyzed with the one-way analysis of variance (ANOVA) or Kruskal-Wallis test according to the data normality homogeneity of variances. All data were shown as mean + SEM (*n* = 3). *P* < 0.05 was considered significantly different. *P < 0.05; **P < 0.01; ***P < 0.001.

In order to assess the frequency of lymphocytes such as CD3^+^CD4^+^ T cells, CD3^+^IFN-γ^+^ Th1 cells, CD3^+^IL-4^+^ Th2 cells, and CD4^+^CD25^+^FoxP3^+^ Treg cells, flow cytometry was performed. In humanized mice (**Figure 8A**), (1) The frequency of lymphocytes in mice vaccinated with MP3RT was remarkably higher than that in mice vaccinated with PBS (*P* = 0.0073, **Figure 8B**); (2) The frequency of CD3^+^CD4^+^ T cells in mice vaccinated with MP3RT was higher than that in mice vaccinated with BCG (*P* = 0.0361, **Figure 8C**); (3) The frequency of CD3^+^IFN-γ^+^ Th1 cells in mice vaccinated with MP3RT was higher than that in mice vaccinated with PBS (*P* = 0.0046, **Figure 8D**) or BCG (*P* = 0.0005, **Figure 8D**); (4) The frequency of CD3^+^IL-4^+^ Th2 cells (**Figure 8E**) or CD4^+^CD25^+^FoxP3^+^ Treg cells (**Figure 8F**) had no significant differences among groups.

In wild-type mice (**Figure S1A**), the frequency of lymphocytes in mice vaccinated with BCG was higher than that in mice vaccinated with PBS (*P* = 0.0219, **Figure S1B**). The frequency of CD3^+^CD4^+^ T cells (**Figure S1C**), CD3^+^IFN-γ^+^ Th1 cells (**Figure S1D**), and CD3^+^IL-4^+^ Th2 cells (**Figure S1E**) had no significant differences among groups. The frequency of CD4^+^CD25^+^FoxP3^+^ Treg cells in mice vaccinated with BCG (*P* = 0.0328) was lower than that in mice vaccinated with PBS (**Figure S1F**).

### MP3RT Stimulated High Levels of Antigen-Specific Antibodies

**Figure 9:**
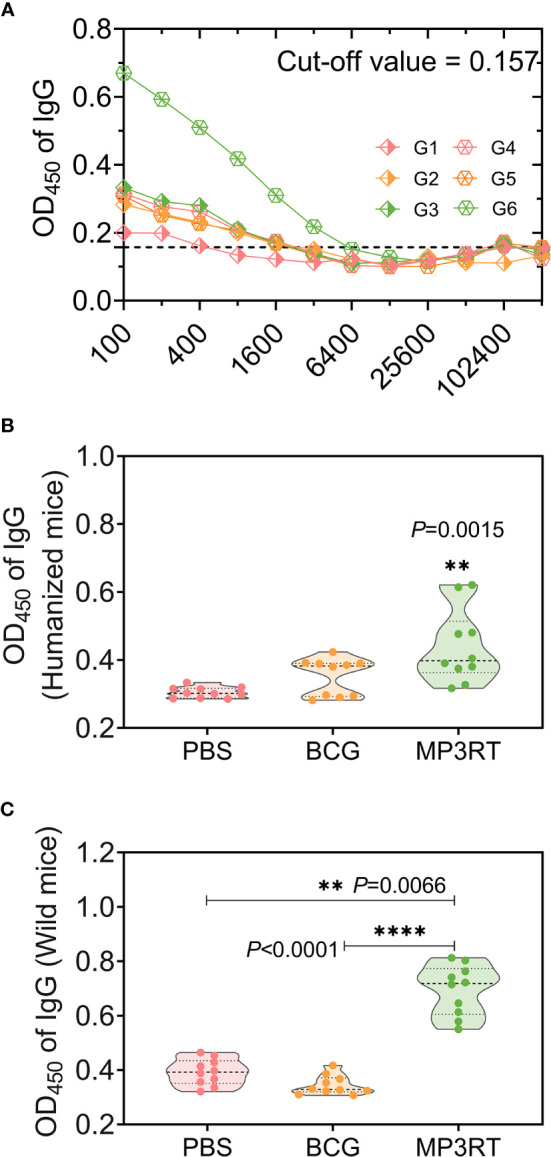
MP3RT-specific IgG antibody. Ninety-one days after the first immunization, the mice in each were killed, and their blood samples were collected for preparing sera. The MP3RT-specific antibodies were detected with ELISA assay. The optimum dilution of IgG **(A)** in sera of humanized or wild-type mice immunized with PBS (G1 or G4 group), BCG (G2 or G5 group), and MP3RT (G3 or G6 group) were determined with a cut-off value. The OD_450_ of IgG antibody in sera of humanized **(B)** or wild-type mice **(C)** was analyzed with a microplate reader. The significant differences of OD_450_ of IgG were analyzed using the one-way analysis of variance (ANOVA) or Kruskal-Wallis test according to the data normality and homogeneity of variances. Data were shown as mean ± SEM (*n* = 10). *P*<0.05 was considered significantly different. **P<0.01; ****P<0.0001.

The level of MP3RT-specific IgG was tested with ELISA. Firstly, the optimal dilution of IgG antibody was determined to be 1:400 with cut-off values of 0.157 (**Figure 9A**). Then, the serum samples were diluted at 1:400, and their OD_450_ values were analyzed with a microplate reader. The results indicated that: (1) In humanized mice, the OD_450_ values of MP3RT-special IgG (*P* = 0.0015, **Figure 9B**) antibody in serum collected from mice vaccinated with MP3RT were significantly higher than those in serum collected from mice vaccinated with PBS or BCG; (2) In wild-type mice, the OD_450_ values of MP3RT-special IgG (**Figure 9C**) antibody in serum collected from mice vaccinated with MP3RT were significantly higher than those in serum collected from mice vaccinated with PBS (*P* = 0.0066) or BCG (*P* < 0.0001).

## Discussion

In this study, we reported a novel peptide-based vaccine, MP3RT. The MP3RT vaccine consisted of six immunodominant epitope peptides from five proteins (Mtb8.4, PPE18, PPE68, RpfA, and TB10.4), which were linked in silico by GGGGS linkers. Mtb8.4 (Rv1174c) and TB10.4 (Rv0288) are low molecular weight T-cell antigens and possible vaccine candidates that induced strong cell-mediated and humoral immune responses against *M. tuberculosis* (26–30). PPE18 (Rv1196) and PPE68 (Rv3873) are members of the *M. tuberculosis* PPE family. PPE18 is a crucial virulence factor for the intracellular survival of *M. tuberculosis* and one of the main components of the M72/AS01E vaccine (31, 32). Recently, Komal Dolasia et al. reported that PPE18 protein inhibited MHC class II antigen presentation (33). However, we found that two peptides (PPE18_115-129_ and PPE18_149-163_) of PPE18 protein could be recognized and presented by splenolymphocytes from humanized mice. PPE68 involves diversifying selection to evade host immunity and stimulating high levels of IFN-γ in PBMCs isolated from TB patients (34, 35). Both are reported to be attractive vaccine candidates for preventive and therapeutic against TB (31, 32, 34, 35). RpfA (Rv0867c), a possible resuscitation-promoting factor, may influence the outcome of reactivation *via* modulating innate immune responses to *M. tuberculosis* (36). It was reported that RpfA induced a high level of IFN-γ and was a TB vaccine candidate (37).

In previous studies, several immunogenic peptides of these five proteins have been identified, such as Mtb8.4_61-69_ (ASPVAQSYL) and Mtb8.4_33-43_ (AVINTTCNYGQ) (38), PPE68_118-135_ (VLTATNFFGINTIPIALT) (34), PPE68_124-156_ (ATNFFGINTIPIALTEMDYFIRMWNQAALAMEV) (39), PPE68_127-136_ (FFGINTIPIA) (40), RpfA_41-60_ (DGEWDQVARCESGGNWSINT) (37), TB10.4_3-11_ (QIMYNYPAM) (41), and TB10.4_20-28_ (GYAGTLQSL) (42). Herein, using the ELISOPT assays, we identified six immunodominant peptides of above five proteins, including Mtb8.4_69-83_ (LRNFLAAPPPQRAAM), PPE18_115-129_ (RAELMILIATNLLGQ), PPE18_149-163_ (AAAMFGYAAATATAT), PPE68_138-152_ (DYFIRMWNQAALAME), RpfA_377-391_ (AYTKKLWQAIRAQDV), and TB10.4_21-35_ (YAGTLQSLGAEIAVE). Interestingly, we found that the peptides Mtb8.4_69-83_, PPE68_138-152_, and TB10.4_21-35_ identified in this study overlapped with the peptides Mtb8.4_61-69_, PPE68_124-156_, and TB10.4_20-28_ previously reported in the literatures (38, 39, 42), respectively. This finding indicates that the IEDB database has unique advantages in predicting Th1-dominant epitopes (8), and it also provides the possibility of using these overlapping epitopes to construct new epitope vaccines in the future.

The most significant difference between MP3RT vaccines and traditional subunit vaccines is that the former are MHC restricted. Therefore, the construction and selection of animal models are the basis for evaluating the protective efficiency of a vaccine. In the current study, we evaluated the protective efficacy of the MP3RT vaccine in humanized C57BL/6 mice (HLA-A11^+/+^DR1^+/+^H-2-β2m^-/-^/IAβ^-/-^) and wild-type C57BL/6 mice, respectively. This humanized mouse model has been successfully used to evaluate the efficacy of an HIV vaccine restricted by the HLA-DR alleles in the Chinese population in our previous study (14). The clinical manifestations of TB patients often show symptom of weight loss. This phenomenon has also been verified in the animal model used in this study. Our research found that regardless of whether it was a wild-type mouse or a humanized mouse, weight loss occurred within one week after *M. tuberculosis* infection. Interestingly, the bodyweight of humanized mice vaccinated with BCG and MP3RT began to recover rapidly after one week, while that of humanized mice in other groups recovered slowly. In particular, several humanized mice, rather than wild-type mice, immunized with PBS died after the *M. tuberculosis* challenge. These data showed at least two conclusions: First, compared with wild-type mice, humanized mice are more susceptible to *M. tuberculosis* infection, which has been observed in our previous study (14); Second, MP3RT vaccine has a considerable protection effect on humanized mice, but the initial immune response induced by MP3RT vaccine does not appear until two weeks after *M. tuberculosis* infection. The delayed activation of the primary immune response might provide a window period for *M. tuberculosis* to develop infection in lung (43). This delay may be caused by the following factors, such as low-dose mycobacteria, low inflammatory response in the lung itself, or suppressing the activation of the primary immune response caused by *M. tuberculosis*.

Similarly, the MP3RT vaccine’s protection was confirmed by CFUs counting experiment and pathological analysis in this study. The results showed that the CFUs in the lungs and livers of humanized mice immunized with MP3RT were significantly reduced and that in the livers of humanized mice and wild-type mice immunized with DNA vaccine were also decreased. The lung is the most common site of initial *M. tuberculosis* infection. The APCs recognize and phagocytose the mycobacteria in the lung and subsequently migrate to the lung-draining lymph nodes to activate T cells (44). Therefore, the lung has essential value in evaluating vaccine protection efficiency. Given this, we further analyzed the lung’s pathological lesions. We found that the lesion area and the number of inflammatory cells in the lungs collected from MP3RT vaccinated mice were significantly smaller than these in PBS immunized mice.

The above data indicated that the MP3RT vaccine had potential immune protection advantages in humanized animal models. We can’t help asking why this vaccine has protective effect only in humanized mice and no protective effect in wild mice? As mentioned above, the most obvious difference between peptides-based vaccines and other subunit vaccines is that the former are designed based on MHC restrictions. It has been reported that the MHC class I or II molecule play an important role in the presentation of particular peptides to cytotoxic T lymphocyte (CTL) or helper T lymphocyte (HTL), respectively (45, 46). The epitopes consisted of MP3RT vaccine were determined by ELISPOT assay based on their significantly higher HLA-DR1 binding affinity, which laid a fundament for the protective effect in humanized C57BL/6 mice (HLA-A11^+/+^DR1^+/+^H-2-β2m^-/-^/IAβ^-/-^) rather than wild C57BL/6 mice (HLA-A11^-/-^DR1^-/-^H-2-β2m^+/+^/IAβ^+/+^). These results also remind us that it is necessary to select a suitable transgenic mouse model in the design, protective evaluation, and immune mechanism exploration of peptides-base vaccines.

Subsequently, we have also explored the immune mechanism behind it. *M. tuberculosis* is an intracellular bacterium, and the host’s elimination and strangulation of *M. tuberculosis* mainly depend on specific T lymphocytes such as Th1 and Th17 cells (2). Previous studies have shown that Th1 cells activate the protective immunity against *M. tuberculosis* infection by secreting IFN-γ and TNF-α to activate the oxidative burst in macrophages, the expression of nitric oxide (NO) synthase 2, and the production of reactive nitrogen intermediates (47, 48). In contrast, Th2 cell response makes the host more susceptible to *M. tuberculosis* infection, and the role of Th17 cells in *M. tuberculosis* infection has not yet been determined (49, 50). This study observed that the MP3RT vaccine stimulated splenocytes producing a high level of IFN-γ and a low level of IL-10 in the humanized mouse model. Furthermore, we also found that the number of IFN-γ^+^ T lymphocytes in spleens collected from mice vaccinated with MP3RT vaccine was more than that in the control group. Unexpectedly, the MP3RT protein stimulated a significantly high level of IFN-γ^+^ T lymphocytes in TB patients, LTBI volunteers and normal controls *in vitro*. However, it is difficult to determine whether the immune response induced by MP3RT is biased towards CD3^+^CD4^+^ T lymphocytes, IFN-γ^+^ T lymphocytes, or CD3^+^CD8^+^ T lymphocytes, because CD3^+^CD8^+^ T lymphocytes can contribute to mycobacteria control by secretion of IFN-γ (51). This doubt was answered in our subsequent flow cytometry experiments. To confirm which type of lymphocyte plays a crucial role in inducing protective effect, we used flow cytometry to classify the T lymphocytes. We found that T lymphocytes’ frequency in the spleens of MP3RT-immunized mice was twice that of the control group. The frequencies of CD3^+^CD4^+^ T lymphocytes and CD3^+^IFN-γ^+^ T lymphocytes in the spleens of humanized mice vaccinated with MP3RT were significantly higher than these of the control group. However, there was no difference in the frequency of CD3^+^IL-4^+^ T lymphocytes and CD4^+^CD25^+^FoxP3^+^ T lymphocytes among the groups. Our data support a positive critical role of MP3RT vaccine in immune protection against *M. tuberculosis* infection depends on up-regulated Th1-type T lymphocytes and down-regulated Treg cells and Th2-type T lymphocytes.

It is generally believed that T cells play an irreplaceable role in eliminating and strangulation of *M. tuberculosis*. Currently, accumulating evidence has suggested that B cells play an important role in resisting *M. tuberculosis*’s respiratory tract infection and inflammation (52, 53). B lymphocytes and T lymphocytes have a wide range of synergy in defending against *M. tuberculosis* infection, and B lymphocytes significantly affect the activation of T lymphocytes (53). To determine the humoral immune response mediated by B cells, we detected the MP3RT-specific IgG titers in serum samples. The results revealed that the MP3RT vaccine induced significantly higher levels of IgG antibody in humanized or wild-type mice.

As exploratory research, this study has some unavoidable shortcomings. First, the epitopes of the MP3RT vaccine were initially designed to bind human HLA-DRB1*01:01 allele instead of HLA-DP, HLA-DQ, HLA-A, HLA-B, and HLA-C alleles, which may lead to the decrease of recognition efficiency of MP3RT vaccine for other alleles. Second, the MP3RT vaccine was composed of Th1 cell epitopes rather than CTL or B cell epitopes, which may weaken this vaccine’s protection efficiency. Third, only MP3RT-specific IgG antibody levels were detected but not subtypes such as IgG, IgG2b, and IgG2c antibody levels, which may have missed a strong evidence for elucidating the immune mechanism of MP3RT. Finally, limited to the types of available transgenic animal models, the MP3RT vaccine contains only HLA-DRB1*01:01 binding epitopes and evaluated only on humanized C57BL/6 mice with HLA-A11^+/+^DR1^+/+^H-2-β2m^-/-^/IAβ^-/-^. In future research, we will further enrich the types of epitopes that constitute MP3RT vaccine and verify them on more transgenic animal models.

## Conclusion

In summary, these data suggested that MP3RT vaccination elicits significant protection against *M. tuberculosis* infection in humanized mice rather than wild-type mice. The potential mechanism of the immune protection depends on MP3RT-specific immune response by triggering the activation of CD3^+^CD4^+^ T lymphocytes and CD3^+^IFN-γ^+^ T lymphocytes characterized by producing a high level of IFN-γ and IgG antibody. Our research once again proves the role of Th1 epitopes in the fight against TB and the importance of vaccine construction, which will provide a basis for future vaccine design.

## Data Availability Statement

The original contributions presented in the study are included in the article/**Supplementary Material**. Further inquiries can be directed to the corresponding author.

## Ethics Statement

The clinical investigation related to PBMCs isolation was approved by the Medical Ethics Committee of the 8th Medical Center of Chinese PLA General Hospital (Approved Number: 2018ST011). The patients/participants provided their written informed consent to participate in this study. The animal study was reviewed and approved by Animal Ethical Committee of the 8th Medical Center of Chinese PLA General Hospital (Approved Number: 309201808171015). Written informed consent was obtained from the owners for the participation of their animals in this study.

## Author Contributions

Conceptualization: XW, and WG. Methodology: WG, YL, JM, ZJ, YX, LW, JW, YZ, and SS. Data analysis: WG, and JM. Software: WG. Writing original manuscript: WG. Review and revising manuscript: XW, and WG. Funding acquisition: WG, and YZ. All authors contributed to the article and approved the submitted version.

## Funding

This study was funded by the National Natural Science Foundation of China (Grant No. 81801643), Beijing Municipal Science & Technology Commission (Grant No. Z181100001718005 and 19L2152), Chinese PLA General Hospital (Grant No. QNC19047), National Project of Infectious Diseases (Grant No. 2017ZX10304402003007, 2012ZX10003008002, 2018ZX10731301005001, and AWS17J015).

## Conflict of Interest

The authors declare that the research was conducted in the absence of any commercial or financial relationships that could be construed as a potential conflict of interest.

## Supplementary Material

Supplementary Figure 1 The frequency of lymphocytes, CD3^+^CD4^+^ T cells, CD3^+^IFN-γ^+^ T cells, CD3^+^IL-4^+^ T cells, and CD4^+^CD25^+^FoxP3^+^ Treg T cells in wild-type mice. The splenocytes suspension was prepared, and the frequency of lymphocytes **(A, B)**, CD3^+^CD4^+^ T cells **(A, C)**, CD3^+^IFN-γ^+^ T cells **(A, D)**, CD3^+^IL-4^+^ T cells **(A, E)**, and CD4^+^CD25^+^FoxP3^+^ regulatory T cells **(A, F)** was quantified with BD IntraSure™ kit. The differences in the frequency of cells among PBS, BCG, and MP3RT groups were analyzed with the one-way analysis of variance (ANOVA) or Kruskal-Wallis test according to the data normality homogeneity of variances. All data were shown as mean + SEM (*n* = 3). *P*<0.05 was considered significantly different. *, *P*<0.05.

## Abbreviations

APC, antigen-presenting cell; BCG, Bacille Calmette-Guérin; CFA, complete Freund’s adjuvant; COVID-19, Corona Virus Disease 2019; ELISA, enzyme-linked immune sorbent assay; ELISPOT, enzyme-linked immunospot; FBS, fetal bovine serum; H&E, hematoxylin and eosin; IEDB, Immune Epitope Database; IFA, incomplete Freund’s adjuvant; IFN-γ, interferon-γ; IL, interleukin; LTBI, latent tuberculosis infection; MHC, major histocompatibility complex; NCBI, National Center for Biotechnology Information; NHFPC, National Health and Family Planning Commission of China; PBMCs, peripheral blood mononuclear cells; PBS, phosphate buffer solution; PHA, phytohemagglutinin; RPMI, Roswell Park Memorial Institute; SEM, standard error of the mean; SFCs, spots forming cells; SI, stimulation index; TB, tuberculosis; Th1, T-helper 1; TNF-α, tumor necrosis factor-α; WHO, World Health Organization; WMA, World Medical Association.
