# Supplementary figures and images for: Peptides-Based Vaccine MP3RT Induced Protective Immunity Against Mycobacterium Tuberculosis Infection in a Humanized Mouse Model
Source: Front Immunol. 2021 Apr 26;12:666290. doi: 10.3389/fimmu.2021.666290 (PMC8108698; doi:10.3389/fimmu.2021.666290)

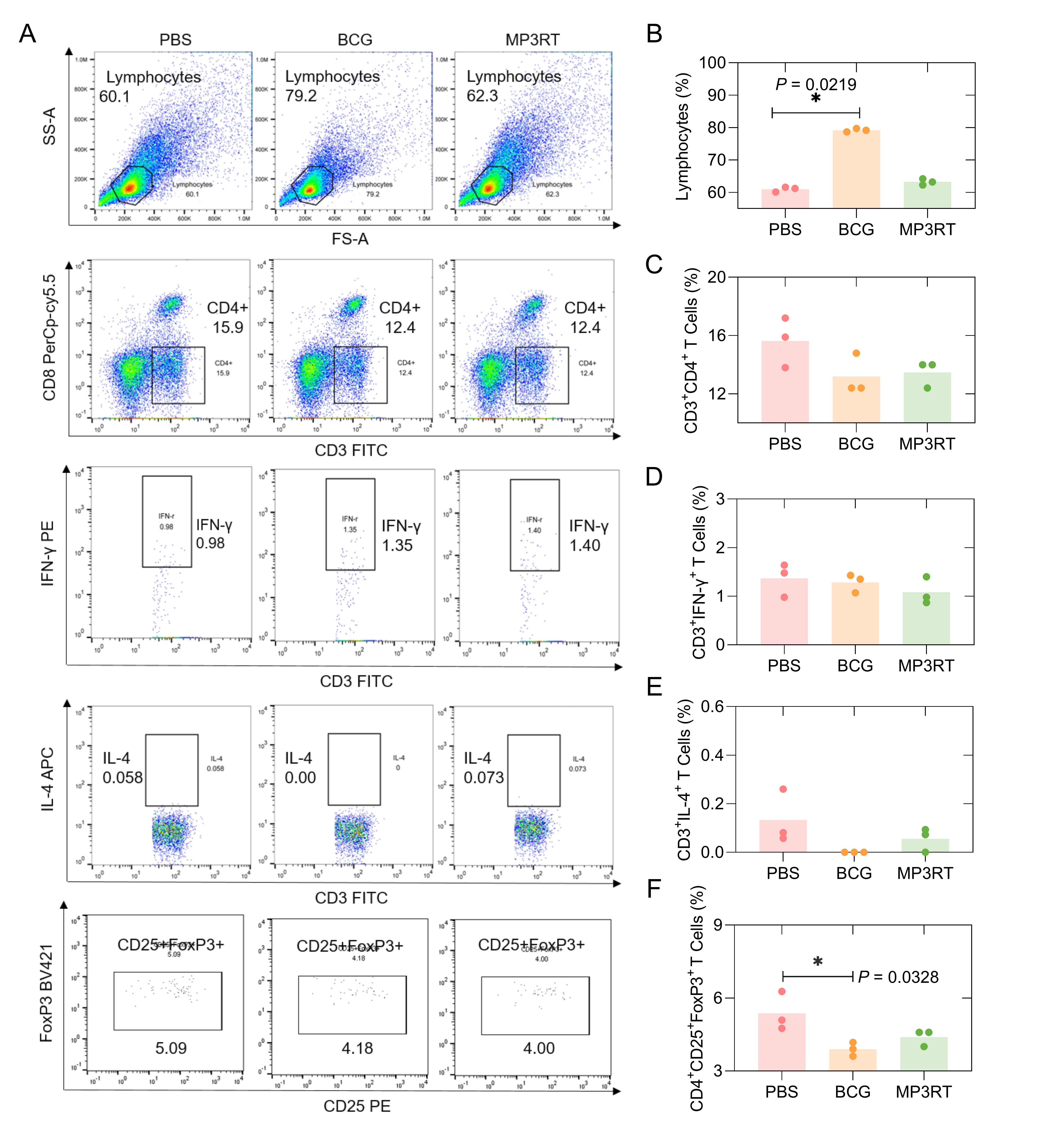

Supplement: Supplementary Figure 1 — The frequency of lymphocytes, CD3+CD4+ T cells, CD3+IFN-γ+ T cells, CD3+IL-4+ T cells, and CD4+CD25+FoxP3+ Treg T cells in wild-type mice. The splenocytes suspension was prepared, and the frequency of lymphocytes (A, B), CD3+CD4+ T cells (A, C), CD3+IFN-γ+ T cells (A, D), CD3+IL-4+ T cells (A, E), and CD4+CD25+FoxP3+ regulatory T cells (A, F) was quantified with BD IntraSure™ kit. The differences in the frequency of cells among PBS, BCG, and MP3RT groups were analyzed with the one-way analysis of variance (ANOVA) or Kruskal-Wallis test according to the data normality homogeneity of variances. All data were shown as mean + SEM (n = 3). P<0.05 was considered significantly different. *, P<0.05. [file Image_1.tif]
